# Mean platelet volume: A versatile biomarker in clinical diagnostics and prognostics

**DOI:** 10.6026/973206300210783

**Published:** 2025-04-30

**Authors:** Garima Anandani, Parth Goswami, Vaishali Bhankhodia, Rushang Dave

**Affiliations:** 1 Department of Pathology, AIIMS Rajkot, Gujarat, India; 2 Department of Pathology, Shantabaa Medical College, Amreli, Gujarat, India

**Keywords:** Mean platelet volume, thrombopoiesis, diagnostic markers, prognostic markers, disease monitoring

## Abstract

The medical field uses mean platelet volume (MPV) measurements as an essential diagnostic and monitoring indicator for diagnosing and following different conditions that include thrombotic and inflammatory diseases with diabetes and other hematological conditions. High mean platelet volume directly correlate with advanced medical conditions in cancer treatments as well as blood-related diseases which include platelet deficiencies. Mean platelet volume increases with inflammatory infections while offering important information about preeclampsia cases in pregnant women. Therefore, the mean platelet volume measurement enables assessment of platelet regulatory processes between production and destruction rate. Thus, this measurement allows healthcare providers to make better decisions regarding patient disease assessment and management.

## Background:

Platelets play a crucial role in the haemostatic process, serving to halt bleeding when necessary. Larger platelets are in an activated state, releasing significant quantities of pro-thrombotic substances that aid in thrombus formation. The size of platelets is quantified by mean platelet volume (MPV). This article examines the relevance of mean platelet volume as a marker for various diseases, including cardiovascular risk (Table 1). It talks about the connection between mean platelet volume and platelet activation and how important it is in thrombotic conditions, which suggests that it could be used to sort people into groups based on their risk. The findings indicate that mean platelet volume is a straightforward and cost-effective biomarker for assessing cardiovascular risks. The study calls for further investigation to standardize mean platelet volume measurements and validate its clinical utility [1, 2]. Shen *et al.* investigated the role of mean platelet volume (MPV) as a biomarker in gastric cancer. The researchers found that elevated pre-operative MPV levels were significantly associated with advanced tumor stages and poorer survival outcomes, suggesting that MPV could serve as a valuable, non-invasive marker for early diagnosis and prognosis in patients with respectable gastric cancer [3].

High mean platelet volume has been connected to not being able to control blood sugar well in people with diabetes mellitus (DM) and a higher risk of vascular problems that come with the disease. The study in question looks into the connection between mean platelet volume and Type 2 DM; specifically how mean platelet volume changes in diabetic patients with and without vascular problems. The results demonstrate a significant elevation of mean platelet volume in individuals with diabetes, showing a strong correlation with fasting glucose, postprandial glucose and HbA1c levels. This suggests that elevated mean platelet volume may either play a role in the development of vascular complications in diabetes or be a consequence of such complications. Mean platelet volume is proposed as a potential marker for assessing vascular risk in individuals with diabetes [4, 5]. In the field of oncology, researchers are investigating increased levels of mean platelet volume as a possible predictive indicator for different types of cancer [6]. Therefore, it is of interest to show mean platelet volume as a versatile biomarker in clinical diagnostics and prognostics.

## Review:

## MPV concept:

Platelet is derived from the hematopoietic lineage via the megakaryocyte. It has been shown more recently that megakaryocytes can be found in the bone marrow or the lungs [7]. Platelets can only live for 5 to 7 days after they are formed and separated from the megakaryocyte. Mean platelet volume gives information about how platelets work and how they are made because bigger platelets are usually younger and more reactive, which means that the bone marrow is actively making platelets [8, 9]. We use automated Hematology analyzers to measure MPV. They follow techniques like impedance or optical methods. The normal range for mean platelet volume can fall between 7.5 and 11.5 fL. Various factors such as age, gender and physiological or pathological conditions can influence variations in mean platelet volume values [10, 11].

## MPV in various pathological conditions:

## Cardiovascular diseases:

## Inflammatory disorders:

[1] Myocardial infarction and stroke:

The process of thrombus formation utilizes platelets. In myocardial infarction and stroke-like diseases, platelets are in their active form. They are large, contain more granules and have a greater propensity to aggregate. Therefore, they are associated with the pathophysiology of myocardial infarction and stroke [12, 13]. Studies have shown that patients with acute myocardial infarction have significantly higher mean platelet volume values compared to those with stable angina or in control groups. Therefore, we could also use the mean platelet volume as a predictive marker for acute coronary syndromes [14].

[2] Atherosclerosis:

In atherosclerosis, the lumen of arteries becomes narrower. Complete obstruction leads to infarction. In pathophysiology, it starts with the endothelial injury. Following the endothelial injury, contact activates platelets, causing them to secrete more prothrombotic and proinflammatory substances [15].

[3] Acute coronary syndrome:

Cardiovascular diseases use elevated mean platelet volume for risk stratification and prognosis. Higher mean platelet volume values have been associated with worse outcomes in these patients. For instance, elevated mean platelet volume is linked to higher mortality rates in patients with acute coronary syndromes. It is considered an independent risk factor for poor prognosis [1, 16].

## Inflammatory disorders:

In inflammatory disorders, there is an increase in MPV. Higher mean platelet volume means reactive and larger platelets. Large platelets produce more pro-inflammatory mediators such as cytokines and chemokines. Elevated mean platelet volume levels show an ongoing inflammatory response [2, 17].

[1] Rheumatoid arthritis:

Studies have shown that mean platelet volume values increase during active disease phases. High mean platelet volume value decline with effective anti-inflammatory treatment. This suggests that we can use mean platelet volume as a biomarker to monitor disease activity and therapeutic response in this patient [4, 18].

[2] Inflammatory bowel disease:

Tumor necrosis factor (TNF)-α, interleukin (IL)-6 and IL-12 are released by activated platelets, macrophages and T cells. These cytokines amplify the inflammatory response. So, more immune cells recruit at the site of inflammation, causing more tissue damage. Platelets are also promoting the adhesion of inflammatory cells and migration to the inflamed gut. Increased MPVs are associated with exacerbations of the disease and can work as an indicator of inflammatory burden. Monitoring the mean platelet volume has a beneficial role for evaluating the severity of the disease and providing guidance for treatment [19].

[3] Systemic lupus erythematosus (SLE):

Elevated mean platelet volume is significantly associated with systemic lupus erythematosus. The mean platelet volume values of patients with active lupus are higher than those of remission patients. This highlights the significance of platelets in systemic lupus erythematosus [20].

## Hematology disorders:

[1] Thrombocytopenia:

There are various causes for low platelet count. It can be due to decreased production or increased destruction of platelets. If there is the destruction of platelets, bone marrow becomes stressed to produce more platelets. They are more active and are increasing in size. If mean platelet volume is normal or low, it indicates bone marrow failure [10, 21]. If you don't count pseudo-thrombocytopenia, thrombocytopenia is mostly caused by less platelet production in the bone marrow or more platelet destruction in the bloodstream. When the bone marrow produces fewer platelets, it leads to hypo-productive thrombocytopenia. This can happen due to conditions like leukemia, aplastic anemia, myelodysplastic syndrome (MDS), or the effects of chemotherapy. Other causes include lymphoma, multiple myeloma, cancer metastasis and megaloblastic anemia. On the other hand, excessive platelet destruction, also known as peripheral consumption, can also cause thrombocytopenia. Immune thrombocytopenic purpura (ITP), an autoimmune condition, is a common example where platelets are rapidly destroyed in the bloodstream, leading to bleeding problems. Various diagnostic techniques have been suggested to determine if a patient's low platelet count is due to reduced production or heightened destruction [22]. The most reliable way to differentiate between hypo-productive and hyper-destructive causes of thrombocytopenia is through bone marrow examination. Nevertheless, this method is invasive, painful, time-consuming, uncomfortable, costly and not very patient-friendly [23, 24]. Also, earlier research has shown that platelet-associated immunoglobulin G (PAIgG) autoantibodies play a role in finding platelet glycoproteins that can be used to diagnose and treat ITP. Nevertheless, detecting antibodies alone is not a reliable diagnostic method. The primary limitation of this method is its low sensitivity; some individuals may not have detectable antibodies when diagnosed. In addition, anti-platelet antibodies can be found in other conditions besides ITP. Therefore, we currently advise against testing plasma for antibodies. Therefore, there is a necessity for a new non-invasive method to diagnose thrombocytopenia [25]. The hardest part about diagnosing ITP is telling it apart from other conditions that cause low platelet counts, since they may show up similarly but need very different treatments. Consequently, having a diagnostic tool that effectively identifies whether a patient's thrombocytopenia is due to ITP or decreased platelet production is crucial. Mean platelet volume can assist in differentiating ITP from thrombocytopenia due to hypoproduction. It offers significant benefits since it is nonintrusive, straightforward, rapid, cost-effective, easy to execute, dependable and typically produced by automated cell counters. It reflects the mean size of platelets in the bloodstream, offering critical insights into megakaryocyte activity. A high mean platelet volume suggests elevated platelet production, whereas a low mean platelet volume signifies reduced platelet production. In ITP, the bone marrow produces a higher quantity of new platelets as a reaction to the heightened destruction of platelets in the spleen. These newly generated platelets, which are larger and younger than the typical ones, are then released into the bloodstream, leading to an elevated mean platelet volume in ITP. Consequently, mean platelet volume may assist in differentiating ITP from hypo-productive thrombocytopenia [22].

[2] Myeloproliferative neoplasm:

MPV is high in essential thrombocythemia (ET) and primary myelofibrosis (PMF). There is overproduction of platelets. These abnormal platelets are large and more immature, suggestive of high MPV. Monitoring mean platelet volume in these patients can help assess disease progression and response to treatment [26].

[3] Thalassemia:

Hemolysis and ineffective erythropoiesis lead to bone marrow stress. Therefore, the production of platelets increases. Monitoring mean platelet volume can help evaluate hematopoietic stress and the effectiveness of treatments [27].

[4] Iron deficiency anemia:

Iron deficiency anemia, a common type of anemia, also impacts MPV. Studies show that patients with iron deficiency anemia usually have lower MPV. This decrease is due to impaired platelet production in the iron-deficient bone marrow. Treating iron deficiency anemia usually normalizes MPV, making it a useful marker for monitoring the response to iron supplementation [28].

## Infectious diseases:

[1] Bacterial infection:

In bacterial infections, mean platelet volume often rises due to increased inflammation. Larger platelets, shown by high MPV, are more active and help the body defend by releasing inflammatory substances. Studies show that patients with sepsis, a severe bacterial infection, often have higher mean platelet volume values, which correlate with disease severity and prognosis [29, 30].

[2] Viral infection:

Monitoring mean platelet volume in sepsis can help identify patients at higher risk of complications. Viral infections also affect MPV, though patterns vary. For instance, dengue causes thrombocytopenia due to various factors and in response, the bone marrow produces larger cells, increasing mean platelet volume [31, 32]. In critical COVID-19 patients, platelet indices like mean platelet volume are linked to inflammation and can predict outcomes. These indices play a significant role in blood clotting and the immune system, making them valuable for assessing severity and mortality risk in severe COVID-19 cases [33].

[3] Parasitic infection:

When someone has a parasitic infection, like malaria, mean platelet volume is high, which means that more platelets are being activated and changing because of the parasite. Mean platelet volume can help diagnose and monitor malaria, providing insights into infection severity and treatment effectiveness [34].

[4] Fungal infection:

In fungal infections, especially invasive ones, mean platelet volume changes. Elevated mean platelet volume in these patients indicates active platelet production and activation in response to the infection. Monitoring mean platelet volume can aid in the early detection and management of fungal infections [35].

## Cancer:

In cancer, mean platelet volume has been studied for its role in diagnosing, predicting outcomes and monitoring treatment. Mean platelet volume can be a diagnostic marker in cancer patients. Changes in mean platelet volume values have been seen in many types of cancer. Higher mean platelet volume has been reported in patients with solid tumors, like lung, breast and colorectal cancers. These higher mean platelet volume levels may indicate increased platelet production and activation, which are often linked to cancers [36, 37]. Mean platelet volume is also a predictor of outcomes in cancer. Higher mean platelet volume values are linked to worse outcomes in various cancers. Studies show that high mean platelet volume is associated with advanced disease stages in cancers like gastric, ovarian and colorectal cancers. This connection between high mean platelet volume and poor outcomes might be due to activated platelets promoting tumor growth and spread [38, 39-40]. Monitoring mean platelet volume levels can give insights into how well cancer treatments are working. Changes in mean platelet volume during chemotherapy or radiotherapy may show the patient's response to treatment. If mean platelet volume goes down after treatment works, it could mean that the tumor has shrunk and that the response was good. On the other hand, if mean platelet volume stays high, it could mean that the tumor is not responding to treatment or that the disease is getting worse [41] Platelets are crucial in cancer progression and spread. Activated platelets can release factors that support tumor growth and spread. Increased MPV, indicating heightened platelet activation, could potentially correlate with more aggressive cancer and increased metastasis risks. Understanding mean platelet volume and platelet activation in cancer can help develop new treatments targeting these interactions [42].

## Diabetes:

Individuals with type 2 DM (T2DM) face an increased risk of cardiovascular disease (CVD), with both microvascular and macrovascular complications linked to elevated blood sugar levels. Bad cardiovascular events are closely linked to insulin resistance (IR). The triglyceride-glucose index (TyG), which is made up of fasting triglycerides (mg/dL) and fasting plasma glucose (mg/dL)/2, is seen as a new way to measure IR. Recently, multiple studies have demonstrated that TyG is related to CVD, particularly arterial issues, in individuals with T2DM and pre-diabetes. Cardiovascular complications linked to T2DM often begin to develop during the pre-diabetes stage. Pre-diabetes, which is considered a precursor to diabetes, is associated with a higher risk of cardiovascular diseases compared to normal blood glucose levels. Platelets play a key role in these complications. When a blood vessel is injured, activated platelets release substances that trigger inflammation, blood clot formation and the development of atherosclerosis [43]. In T2DM, platelets show changes in their signaling pathways, leading to increased activity. Because they are working harder, they are more sensitive to small changes in their environment. This makes them make more highly reactive platelets by forming them faster [44, 45]. Poor blood sugar control and higher mean platelet volume are significant factors that contribute to both small and large blood vessel complications in T2DM. Changes in platelet indices are common in diabetes, especially T2DM and are associated with a higher risk of heart and blood vessel problems. In diabetic patients, mean platelet volume is usually higher. This is important because larger platelets are more active and more likely to form clots, which can lead to heart issues [46]. Higher mean platelet volume in diabetic patients is linked to a greater risk of heart attacks and strokes. High blood sugar levels in diabetes cause platelets to become more active and stick together, increasing MPV. Poor blood sugar control is associated with higher mean platelet volume levels. Patients with high HbA1c levels, indicating chronic high blood sugar, often have higher MPV, showing that long-term high blood sugar increases platelet activity [47]. The exact mechanisms that contribute to the rise in mean platelet volume in individuals with pre-diabetes and T2DM are not completely understood. One potential explanation is that insulin resistance is a central factor in various metabolic disorders associated with heightened platelet activity. Insulin resistance can stop insulin from doing its normal job on platelets, like stopping them from sticking together and increasing the production of nitric oxide (NO) by turning on NO synthase. This process increases the levels of cyclic adenosine monophosphate (cAMP) in platelets. Through advanced glycation end-product receptors, high blood sugar can also tell the liver and neutrophils to make thrombopoietin, which makes platelets bigger. Platelets that are bigger are more metabolically and enzymatically active, releasing more serotonin and making more thromboxane A2, which can lead to problems with blood vessels. In individuals with prediabetes and diabetes, an increase in mean platelet volume has been associated with arterial stiffness. Mean platelet volume is often used as an indicator of platelet activation and vascular health. Activated platelets release substances that affect blood vessel function, such as reactive oxygen species (ROS), which reduce NO availability and growth factors, which encourage smooth muscle cell growth. Too many platelets can also cause too many matrix metalloproteinases (MMPs) to work, which breaks down elastic fibres and makes arteries less flexible. These factors highlight the connection between platelet activation, atherosclerosis and cardiovascular risks in individuals with pre-diabetes and diabetes [43, 48]. When comparing people with impaired glucose tolerance and T2DM to people with normal glucose tolerance, myocardial deformation indices are important. The Global Longitudinal Strain (GLS), a key measure for assessing heart muscle function, was found to be significantly higher in individuals with increased MPV. Mean platelet volume emerged as the second most important predictor of GLS, highlighting its potential to detect early heart muscle damage. Patients with ischemic or idiopathic cardiomyopathy showed higher mean platelet volume levels, which were linked to impaired left ventricular function. This could be due to blood pooling in an enlarged heart chamber and weak heart contractions, which may activate platelets and raise mean platelet volume levels. Additionally, people with coronary artery disease (CAD) had higher mean platelet volume compared to healthy individuals and the risk of CAD was greater in those with elevated mean platelet volume than those with lower levels [43].

## Pregnancy-related disorders:

[1] Preeclampsia:

In preeclampsia, mean platelet volume is often higher. This rise in mean platelet volume may be due to increased platelet activity and use. Larger, more reactive platelets contribute to the higher clotting risk seen in preeclampsia [49]. High mean platelet volume levels can help detect preeclampsia early. Research shows that mean platelet volume is much higher in women with preeclampsia compared to those with normal blood pressure during pregnancy. The severity of preeclampsia is also linked to higher mean platelet volume levels, with more severe cases showing worse outcomes for both mother and baby [50].

[2] Gestational diabetes mellitus (GDM):

Researchers have found changes in mean platelet volume in women with GDM. Higher mean platelet volume in GDM suggests more platelet activity, which could lead to a higher risk of blood clots and vascular problems. Studies show that higher mean platelet volume is linked to poor blood sugar control in pregnant women with GDM. Keeping track of mean platelet volume can help evaluate how well treatments are managing blood sugar levels [51].

[3] Intrauterine growth restriction:

Higher mean platelet volume in women with intrauterine growth retardation (IUGR) may mean that the placenta isn't working right and that the baby isn't getting enough oxygen. Active platelets are involved in IUGR by affecting blood flow to the placenta. mean platelet volume can help find pregnancies that might have IUGR; higher mean platelet volume means there might be a problem with the placenta, so the pregnancy needs to be closely watched [52].

## Conclusion:

The importance of mean platelet volume as a useful marker in medicine is shown. Mean platelet volume gives valuable information about platelet function and activation, helping doctors diagnose and predict outcomes for many health conditions. Mean platelet volume is a reliable marker that improves our understanding of different diseases, making diagnosis more accurate and aiding in treatment decisions. The role of mean platelet volume in new medical situations is relevant to strengthen its use in personalized patient care.

## Author contributions:

Parth Goswami and Garima Anandani conducted the literature review. Rushing Dave drafted the manuscript. Parth Goswami, Garima Anandani and Vaishali Bhankhodia contributed in reviewing and supervising the manuscript. All authors contributed to the article and approved the submitted version.

## Ethical statement:

The authors confirm that we have adhered to the ethical policies of the journal. No identification details of the patient are shared.

## Funding:

No funds were received or used.

## Figures and Tables

**Table 1:** Common associations of altered mean platelet volume (MPV)

**High MPV**	**Low MPV**
Atrial fibrillation	Ulcerative colitis
Certain malignancies	Thrombocytosis
Stroke	Inflammatory bowel disease
Heart disease	Chemotherapy
High blood pressure	Crohn's disease
Diabetes	Splenomegaly
Vitamin D deficiency	Iron deficiency and aplastic anemia
Hypothyroidism and hyperthyroidism	

## References

[1] Chu S.G (2010). Journal of Thrombosis and Haemostasis..

[2] Kisacik B (2008). Joint Bone Spine..

[3] Shen X.M (2016). Oncol Lett..

[4] Yazici M (2009). Platelets..

[5] Kodiatte T.A (2012). J Lab Physicians..

[6] Wu Y.Y (2019). BMC Cancer..

[7] Lefrancais E (2017). Nature..

[8] Thon J.N, Italiano Jr-J.E (2012). Blood..

[9] Schwertz H (2010). Blood..

[10] Patrizia N (2016). Platelets..

[11] Bath P.M, Butterworth R.J (1996). Blood Coagul Fibrinolysis..

[12] Tsiara S (2003). Clin Appl Thromb Hemost..

[13] Vizioli L (2009). Int J Clin Pract..

[14] Sansanayudh N (2014). Int J Cardiol..

[15] Zhu N (2020). Medicine (Baltimore)..

[16] Martin J.F (1991). Lancet..

[17] Gasparyan A.Y (2011). Curr Pharm Des..

[18] Moghimi J (2017). Pan Afr Med J..

[19] Bambo G.M (2022). PLoS One..

[20] Ekiz O (2014). Angiology..

[21] Bessman JD (1981). Am J Clin Pathol..

[22] Walle M (2023). PLoS One..

[23] Negash M (2016). BMC ematology..

[24] Mowafy N.M (2019). The Egyptian Journal of Hospital Medicine..

[25] Sachs U.J (2019). Hämostaseologie..

[26] Bowles K.M (2005). Clin Lab Haematol..

[27] Ali M (2017). Acta Haematologica..

[28] Subramaniam N (2014). ISRN Hematol..

[29] Vardon-Bounes F (2019). Int J Mol Sci..

[30] Kim C.H (2015). PLoS One..

[31] Ahmad W (2020). Arch Hematol Case Rep Rev..

[32] Mavilla A (2014). Int J Med Sci Public Health..

[33] Cankar H (2022). Journal of Health Sciences and Medicine..

[34] Ladhani S (2002). Br J Haematol..

[35] Klinger M.H, Jelkmann W (2002). Journal of Interferon and Cytokine Research..

[36] Kemal Y (2014). J Obstet Gynaecol..

[37] Mutlu H (2013). Clin Appl Thromb Hemost..

[38] Mammadova-Bach E (2015). Hamostaseologie..

[39] Ferroni P (2014). Haematologica..

[40] Masternak M (2019). Acta Haematologica Polonica..

[41] Omar M (2018). Clinical Respiratory Journal..

[42] Dudiki T (2023). Circulation Research..

[43] Cassano V (2024). Cardiovasc Diabetol..

[44] Lekston A (2014). J Diabetes Complications..

[45] Vazzana N (2012). Thromb Res..

[46] Inoue H (2020). J Diabetes Investig..

[47] Agrawal J (2017). Diabetes and Metabolic Syndrome..

[48] Wang R.T (2011). Platelets..

[49] Sachan R (2021). J Family Med Prim Care..

[50] Thalor N (2019). Hematol Transfus Cell Ther..

[51] Ren Z (2020). Chin Med J (Engl)..

[52] Ciobanu A.M (2021). Medicina (Kaunas)..

